# A mechanistic marker-based screening tool to predict clinical immunogenicity of biologics

**DOI:** 10.1038/s43856-023-00413-7

**Published:** 2023-12-08

**Authors:** Nicole L. Jarvi, Sathy V. Balu-Iyer

**Affiliations:** Department of Pharmaceutical Sciences, University at Buffalo, The State University of New York, Buffalo, NY 14214 USA

**Keywords:** Applied immunology, Immunology, Drug development, Recombinant protein therapy

## Abstract

**Background:**

The efficacy and safety of therapeutic proteins are undermined by immunogenicity driven by anti-drug antibodies. Immunogenicity risk assessment is critically necessary during drug development, but current methods lack predictive power and mechanistic insight into antigen uptake and processing leading to immune response. A key mechanistic step in T-cell-dependent immune responses is the migration of mature dendritic cells to T-cell areas of lymphoid compartments, and this phenomenon is most pronounced in the immune response toward subcutaneously delivered proteins.

**Methods:**

The migratory potential of monocyte-derived dendritic cells is proposed to be a mechanistic marker for immunogenicity screening. Following exposure to therapeutic protein in vitro, dendritic cells are analyzed for changes in activation markers (CD40 and IL-12) in combination with levels of the chemokine receptor CXCR4 to represent migratory potential. Then a transwell assay captures the intensity of dendritic cell migration in the presence of a gradient of therapeutic protein and chemokine ligands.

**Results:**

Here, we show that an increased ability of the therapeutic protein to induce dendritic cell migration along a gradient of chemokine CCL21 and CXCL12 predicts higher immunogenic potential. Expression of the chemokine receptor CXCR4 on human monocyte-derived dendritic cells, in combination with activation markers CD40 and IL-12, strongly correlates with clinical anti-drug antibody incidence.

**Conclusions:**

Mechanistic understanding of processes driving immunogenicity led to the development of a predictive tool for immunogenicity risk assessment of therapeutic proteins. These predictive markers could be adapted for immunogenicity screening of other biological modalities.

## Plain language summary

Protein drugs have a structure that is very similar to proteins in the human body, however there are slight differences. This can result in a person’s immune system having a negative reaction to the drug. Unfortunately, during the early stages of drug development, it is currently difficult to predict which of these potential new drugs might cause this problem. We think that there is a higher chance for undesired immune responses to protein drugs when they cause an increase in the movement of the body’s immune cells from the drug injection site to the lymph nodes. The more immune cells that arrive in the lymph node, the stronger the immune reaction. We developed a method that can predict how likely it is that a protein drug will trigger the unwanted activation and movement of these immune cells. This tool has the potential to improve our ability to predict, and thus avoid, unwanted immune system reactions toward protein drugs. This could shorten the time and cost required to develop new drugs.

## Abstract (web summary)

Jarvi and Balu-Iyer evaluate an immunogenicity screening toolbox in which in vitro assays capture the migratory potential of dendritic cells. Expression of the chemokine receptor CXCR4 on dendritic cells and their migration toward chemokines CCL21 and CXCL12 in the presence of therapeutic protein correlates with their clinical immunogenicity.

## Introduction

Anti-drug antibodies (ADA) formed against therapeutic proteins can majorly impact product safety and efficacy depending on their binding or neutralizing activity^1–5^. Immunogenicity can even cause the termination of a development program^6,7^, thus immunogenicity risk assessment of therapeutic protein candidates is critical. ADA development against therapeutic proteins is the consequence of an adaptive immune response driven by antigen-specific interactions between immune cells, namely dendritic cells, T cells, and B cells. Humoral responses driven by B cells can be captured by ELISPOT analysis after in vivo administration of protein, and IgM, but not IgG, responses were predicted in an in vitro lymph node model^8–10^. The T-cell dependent immune response is the rationale for T-cell based immunogenicity risk assessment approaches, including T-cell proliferation/cytokine production assays, in silico screening of T-cell epitopes (T helper and/or Tregitopes), and T-cell epitope presentation in MHC-associated peptide proteomics (MAPPs)^11,12^. However, current gaps in the ability of immunogenicity risk assessment to predict clinical immunogenicity outcomes reveal the insufficiency of available methods^13,14^. T-cell activation and differentiation during the adaptive immune response requires strong, prolonged signals; and with a T-cell-focused approach, the early steps in the innate immune response are overlooked.

A key step in the early phases of immune response is the migration of dendritic cells into T-cell areas of lymphoid compartments to mediate naive T-cell activation^15^. This phenomenon is most pronounced upon subcutaneous (SC) administration of protein antigen where dermis-derived dendritic cells (DC), continuously surveying the skin, capture antigens, and migrate to nearby draining lymph nodes (DLN)^16^. Interest in SC delivery of therapeutic proteins has continued to rise due to the advantages of improved cost, convenience, and compliance compared to intravenous (IV) injection; however, the SC route introduces unique immunogenicity challenges compared to intravenous delivery^17–19^. Several therapeutic proteins and monoclonal antibodies (mAb) demonstrated enhanced immunogenicity by SC administration when directly compared to IV^19–26^. Upon SC administration of protein antigen, dermis-derived DCs migrate into the SC space and toward the initial lymphatics; upon arrival in the DLNs they migrate to the subcapsular sinus and into the T-cell area to reach T cells for antigen presentation^15,27,28^. The antigen-loaded migratory DCs, which have matured along the migration path, strongly induce CD4^+^ T-cell activation upon arriving in the lymph node^15,27^. When the arrival of migratory DCs in the DLN is prevented, by surgical removal of the injection site one-hour post-injection, the number of T follicular helper cells, germinal center B cells, and IgG-secreting cells in the DLN are reduced^29^. Thus, migratory skin-DCs and not lymph node-resident DCs were responsible for sustaining germinal center B-cell responses and T follicular helper cell expansion^29^.

The major driver of DC migration under inflammatory and homeostatic conditions is the receptor-ligand interaction between CCR7 on DCs and its ligands CCL19 and CCL21^28,30^, although skin DC migration is not completely inhibited in *CCR7*^*-/-*^ mice^31^. Expression of CCL21 on the lymphatic endothelium and on fibroblastic reticular cells in the DLN creates a haptotactic gradient for DC migration^30^. CCR7 expression is essential for the migration of DCs under steady-state conditions where DCs acquire a “semi-mature” phenotype and can induce peripheral tolerance^32,33^. Comparatively, CXCR4 plays a major role in the migration of activated DCs into DLNs, and expression of the associated ligand CXCL12 in lymphatic vessels is upregulated upon antigen exposure^31^. The degree of DC migration and maturation is a function of the context of antigen exposure (e.g., inflammation) and the features of the antigen^15,34^. We hypothesize that a therapeutic protein would upregulate DC migration, for example into the SC injection site and toward the DLNs, in proportion to its immunogenic potential^17,18^. Furthermore, the presence of risk factors (danger signals or adjuvants) in the drug product or injection site could increase skin-derived DC migration^35^. By exposing dendritic cells to therapeutic protein in vitro, induction of migratory potential could be assessed and transformed into a marker for immunogenic risk.

Here, multiple markers of DC migratory potential are assembled to predict immunogenicity risk for therapeutic proteins. The migratory potential of DCs in response to therapeutic protein is captured by CXCR4 expression with markers for DC activation, such as CD40, which initiates DC migration. Also, a transwell assay captures DC migratory potential by simulating migration toward chemokines CCL21 and CXCL12. The readouts of DC migratory potential correlate strongly with clinical immunogenicity incidence for a range of therapeutic proteins.

## Methods

### Materials

Lipopolysaccharides (LPS) from *E. coli* O55:B5, heat-inactivated male type AB human serum, gentamicin sulfate, penicillin/streptomycin 100X solution, and keyhole limpet hemocyanin (KLH, catalog #H7017) were purchased from Sigma-Aldrich (St. Louis, MO). Fluorescently labeled antibodies, compensation beads, cell permeabilization buffer (TNB-1213-L150), brefeldin A 1000X solution, monensin 1000X solution, and live/dead “ghost dye” for flow cytometry were purchased from Tonbo™-a Cytek® Brand (San Diego, CA) and Biolegend (San Diego, CA). CountBright™ Absolute Counting Beads were obtained from Thermo Fisher Scientific (Waltham, MA). Human cytokines (IL-4 and GM-CSF) and chemokines (CCL21/Exodus-2 and CXCL12/SDF-1 isoform α) were purchased from Sino Biological (Wayne, PA). Corning Inc. (Corning, NY) RPMI-1640, EDTA solution, Hank’s balanced salt solution (HBSS), and tissue culture-treated plates (catalog #3548 and #3512) were used for all experiments. 96-well transwell plates (catalog #3384 and #3387) were obtained from Corning Inc. (Corning, NY). Biosimilar research-grade monoclonal antibodies were purchased from Absolute Antibody (Boston, MA) (tocilizumab #Ab00737-10-0, ATR-107 #Ab01293-10-0, and HuA33 #Ab00782-10-0) and Bio X Cell (Lebanon, NH) (adalimumab #SIM0001, trastuzumab #SIM0005, and rituximab #SIM0008). Emicizumab (Hemlibra) was generously provided by WNY BloodCare (Buffalo, NY). Endo-Grade ovalbumin (catalog #LET0028) was purchased from BioVendor (Asheville, NC).

### Culture of human monocyte-derived dendritic cells

Cryopreserved, human leukocyte antigen (HLA)-typed peripheral blood mononuclear cells (PBMC) from anonymized healthy donors were purchased from Cytologics (San Diego, CA). Further information about Cytologics’ quality standards for donor consent and ethical regulation compliance can be found at www.cytologicsbio.com/quality-standards/. Cytologics obtains primary cells via donation in the United States in compliance with applicable federal, state, and local laws, regulations, and guidance. Cells are obtained from donors who are voluntarily participating in a donor program approved by an IRB, FDA, or equivalent regulatory authority. In accordance with FDA regulations or as approved by an IRB, these individuals have either donated their cells or have been reasonably compensated for their time and effort during donation. Strict controls on personal identifiers protect donors. Researchers cannot access any identifying information about donors; we were not provided with donor identification information when purchasing these cells. Donors were distinguished by the last three digits of their lot number (e.g., 919). The age range and HLA genotype of donors are provided in Supplementary Table 1. PBMCs were stored in vapor phase liquid nitrogen until use.

Monocyte-derived dendritic cells (moDC) were cultured from human monocytes as described in the literature^36,37^. First, classical CD14^+^CD16^-^ monocytes were isolated by negative selection on Miltenyi MS columns following the instructions provided in the Classical Monocyte isolation kit (Miltenyi Biotec, Gaithersburg, MD). Monocytes were cultured at 37 °C and 5% CO_2_ for five days in complete media at approximately 5 × 10^5^ cells/mL with 50 ng/mL interleukin-4 (IL-4) and 50 ng/mL granulocyte-macrophage colony-stimulating factor (GM-CSF). The complete media was RPMI-1640 with 10% human serum, 1% Penicillin/streptomycin, 30 μg/mL gentamicin sulfate, and 50 μM 2-mercaptoethanol. Half of the media was changed every two days and replaced with additional complete media containing 100 ng/mL IL-4 and 100 ng/mL GM-CSF. No bacterial contamination of cell cultures was observed by microscopic evaluation; testing for mycoplasma contamination was not performed. On day five, moDCs, considered immature, were harvested and used immediately in immunogenicity screening experiments. DC differentiation was checked by flow cytometry (CD11c^+^HLA-DR^+^DC-SIGN^+^CD14^low^) (Supplementary Fig. 1).

### Dendritic cell markers for immunogenicity prediction

Immature moDC were cultured at 37 °C and 5% CO_2_ for 24 h in complete media with 5 μg/mL therapeutic protein in the presence of 100 pg/mL LPS to promote antigen uptake and processing by DCs (without promoting full maturation). The final concentrations of therapeutic protein and LPS were chosen based on preliminary dose-response experiments performed with KLH. Immature moDC cultured in complete media represented the untreated control. Immature moDC were also treated with 1 μg/mL LPS to confirm their ability to mature. For intracellular cytokine staining, protein transport inhibitors brefeldin A (3 μg/mL) and monensin (2 μM) were added five hours before harvesting. MoDC were stained for CD11c (FITC or PerCP-Cy5.5, clone 3.9), HLA-DR (APC-Cy7, clone L243), CD40 (PE, clone G28.5), CXCR4 (APC, clone 12G5), and live/dead (Violet450 ghost dye). Then cells were fixed in 2% buffered formalin phosphate and permeabilized for intracellular staining of IL-12p40 (PerCP-Cy5.5 or FITC, clone C11.5). Stained moDC were stored in MACS buffer at 4 °C.

### Flow cytometry analysis

Ten-thousand events per sample were acquired in FACSDiva on the BD LSRFortessa. Unstained and single-stained samples were prepared to facilitate compensation. Fluorescence-minus-one (FMO) samples were prepared to set positive gates for IL-12p40 PerCP-Cy5.5 and CXCR4 APC by staining cells with all fluorophore-antibody pairs except the one of interest. In FlowJo, the main cell population was gated by forward scatter (FSC) vs side scatter (SSC), then single cells were gated by forward scatter height (FSC-H) vs forward scatter area (FSC-A), and live cells were gated by low viability dye expression (Supplementary Fig. 1a). MoDC were gated as CD11c^+^HLA-DR^+^ and represented the majority (≥ 75%) of live cells present. Within CD11c^+^HLA-DR^+^ moDC the frequencies of CXCR4^+^, CD40^high^, and IL-12p40^+^ cells were determined (Supplementary Fig. 1b, c).

### Dendritic cell migration in a transwell assay

The migration of immature moDC toward therapeutic protein was tested in a transwell assay by creating a concentration gradient of therapeutic protein and chemokines across the transwell insert. Therapeutic protein formulations were prepared in serum-free media with 100 ng/mL of each CCL21 and CXCL12 and added to the bottom chambers of a 96-well transwell plate. Control conditions included media with and without chemokines. The upper chambers were filled with immature moDC (1.6 × 10^5^ cells/mL) in serum-free media containing therapeutic protein. Concentrations of therapeutic protein across the upper and lower chamber were 10 to 50 μg/mL or 100 to 1000 μg/mL. Transwell plates were incubated at 37 °C and 5% CO_2_ for 2.5 h to allow migration of moDC into the lower chamber.

The transwell insert tray was removed, inverted, and tapped on absorbent paper to discard media from the upper chambers. At the same time, the plate was centrifuged and the supernatant was discarded then wells were filled with washing buffer (10 mM EDTA in HBSS). The insert tray was returned to the plate, and the upper chambers were filled with washing buffer. The plate was incubated for 10 minutes to allow dissociation of cells attached to the underside of the insert. The insert tray was then discarded. The plate was centrifuged and the supernatant was discarded. Cells were stained with anti-human DC-SIGN PE (clone 9E9A8) in MACS buffer for 30 min. The plate was centrifuged and the supernatant was discarded. Cells were fixed in 2% buffered formalin phosphate and CountBright counting beads were spiked into each well to facilitate cell counting. Migrated moDC were counted in the transwell plate on the Miltenyi MACSQuant Analyzer 10 using the 96 chill rack and autosampler function. Percent migrated was calculated as (number of migrated moDC/number of plated moDC) x 100%. The migration index was calculated by normalizing the percent migrated for therapeutic protein+CCL21 + CXCL12 against the percent migrated for media+CCL21 + CXCL12.

### Statistics and reproducibility

Within each experiment, treatments were tested in triplicate, and two independent experiments were performed for at least four donors for reproducibility. Flow cytometry data was analyzed in FlowJo v10.7. All graphical and statistical analysis was performed in GraphPad Prism v9. Statistical significance of flow cytometry results was determined by unpaired student’s *t*-test or one-way ANOVA with Dunnett’s multiple comparisons test at significance level *α* = 0.05.

### Reporting summary

Further information on research design is available in the Nature Portfolio Reporting Summary linked to this article.

## Results

### CXCR4 is upregulated on dendritic cells by immunogenic therapeutic proteins

**Fig. 1:**
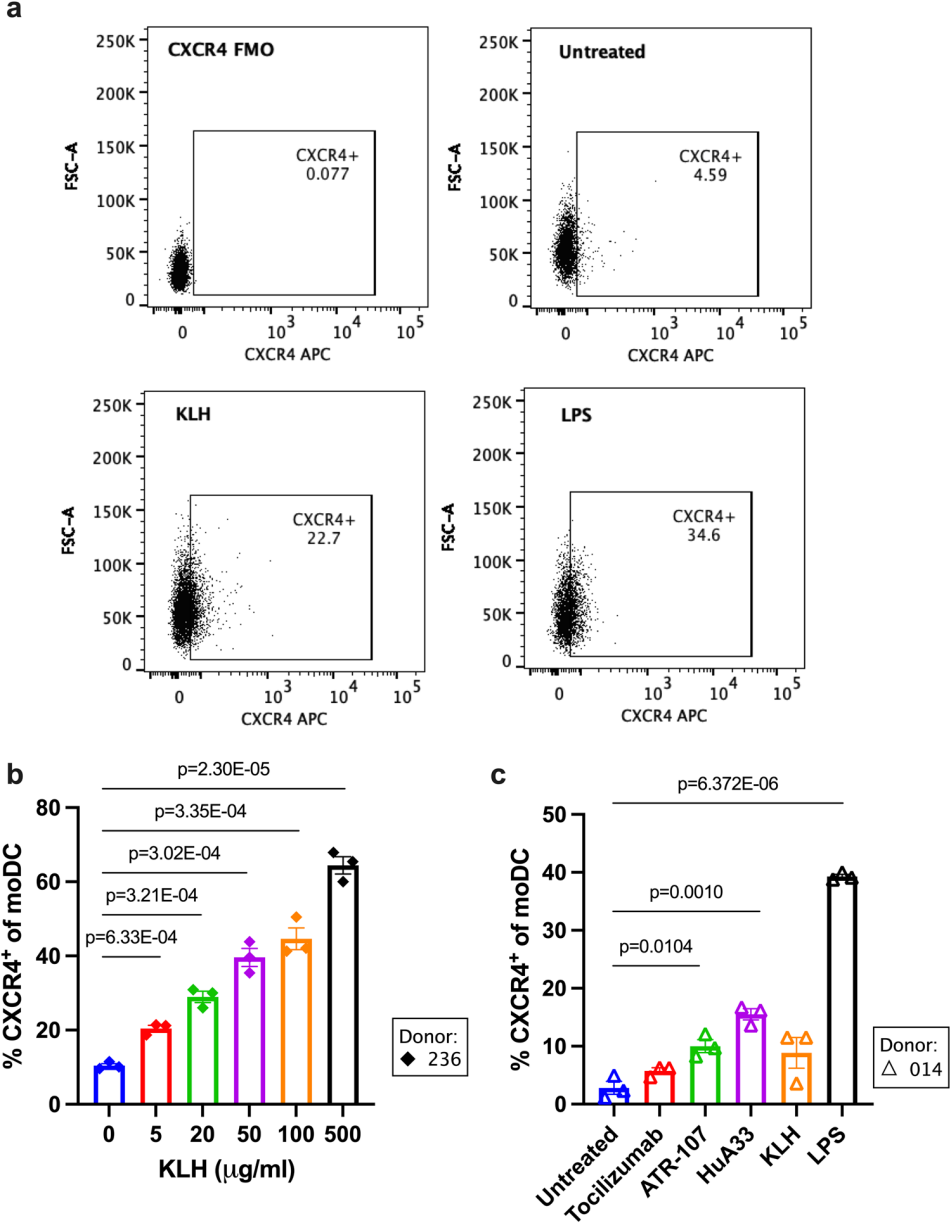
CXCR4 is upregulated on dendritic cells by therapeutic proteins in proportion to their immunogenic potential. **a** Representative flow cytometry dot plots for CXCR4^+^ moDC (CD11c^+^HLA-DR^+^) after 24 h stimulation of moDC with media (“untreated”), LPS 1 μg/mL (“LPS”), or KLH 5 μg/mL + LPS 100 pg/mL (“KLH”). “CXCR4 FMO” represents a control sample stained with all antibody-fluorophore pairs except CXCR4 APC. Supplementary Fig. 1 shows the gating strategy for moDC (CD11c^+^HLA-DR^+^). **b** Frequency (%) of CXCR4^+^ moDCs (CD11c^+^HLA-DR^+^) for a representative donor (#236) after 24 h stimulation with media or KLH (5, 20, 50, 100, or 500 μg/mL) plus 100 pg/mL LPS. **c** Frequency (%) of CXCR4^+^ moDCs (CD11c^+^HLA-DR^+^) for a representative donor (#014) after 24 h stimulation with media (untreated), LPS (1 μg/mL), or 5 μg/mL tocilizumab, ATR-107, HuA33, or KLH (with 100 pg/mL LPS as in **a**). In **b**, **c**, treatments were tested in triplicate, each dot represents a technical replicate and error bars are mean ± SEM. Statistical significance between treatments was determined by a two-tailed unpaired student’s *t*-test (*α* = 0.05). Exact *p*-values are reported unless *p* > 0.05, i.e., not significant. Estimated effect sizes (Cohen’s *d*) and results for one-way ANOVA with Dunnett’s multiple comparisons test are in Supplementary Data.

We sought to determine if CXCR4 was upregulated on dendritic cells by therapeutic proteins in proportion to their immunogenic potential. Monocyte-derived dendritic cells (moDC) were stimulated with therapeutic proteins for 24 h followed by flow cytometry analysis for CXCR4 expression (Fig. 1a and Supplementary Fig. 1). MoDCs generated in vitro display similar phenotypic characteristics to dermal DCs, including expression of CD1a, CD11b, CD101, and Factor XIIIa^38^. Also, the moDCs used here are equivalent or similar in phenotype to those used in other immunogenicity risk assessment strategies, such as MAPPS and DC internalization assays^12,39–41^. Keyhole limpet hemocyanin (KLH), a highly immunogenic protein, was used as the positive control which is common for in vitro immunogenicity risk assessment^42,43^. Twenty-four-hour exposure to increasing concentrations of KLH induced CXCR4 expression on moDC in a dose-dependent manner. The frequency of CXCR4^+^ moDCs in a representative donor (#236) increased from 20.4% at 5 μg/mL KLH to 64.4% at 500 μg/mL KLH, where the CXCR4^+^ moDC population was 10.4% in media alone (“untreated”) (Fig. 1b). The 5 μg/mL dose was thus sufficient to induce a significant increase in CXCR4 expression over the untreated control (*p* = 0.0147). This dose was used to compare the ability of therapeutic proteins with varying immunogenic potential to upregulate CXCR4: ATR-107, HuA33, and tocilizumab. Tocilizumab has very low ADA incidence across patient populations ranging from 0.8–2%, and HuA33 and ATR-107 demonstrated high ADA incidence in phase I clinical trials (73 and 76%, respectively)^44–46^. Target and structure are presented in Supplementary Table 2. For a representative donor (#014), the mean frequency of CXCR4^+^ moDC was found to be 2.8% for untreated cells, and in response to tocilizumab, the frequency of CXCR4^+^ moDC increased to 5.7% (ns) (Fig. 1c). Comparatively, in response to ATR-107, HuA33, or KLH, the mean frequency of CXCR4^+^ moDC increased significantly to 10%, 15.5%, and 8.9% respectively (*p* = 0.008, <0.0001, 0.032) (Fig. 1c). Thus, CXCR4 was upregulated on moDC by monoclonal antibodies in accordance with their immunogenic potential.

### CXCR4 upregulation correlates with maturation/activation marker upregulation by immunogenic therapeutic proteins

**Fig. 2:**
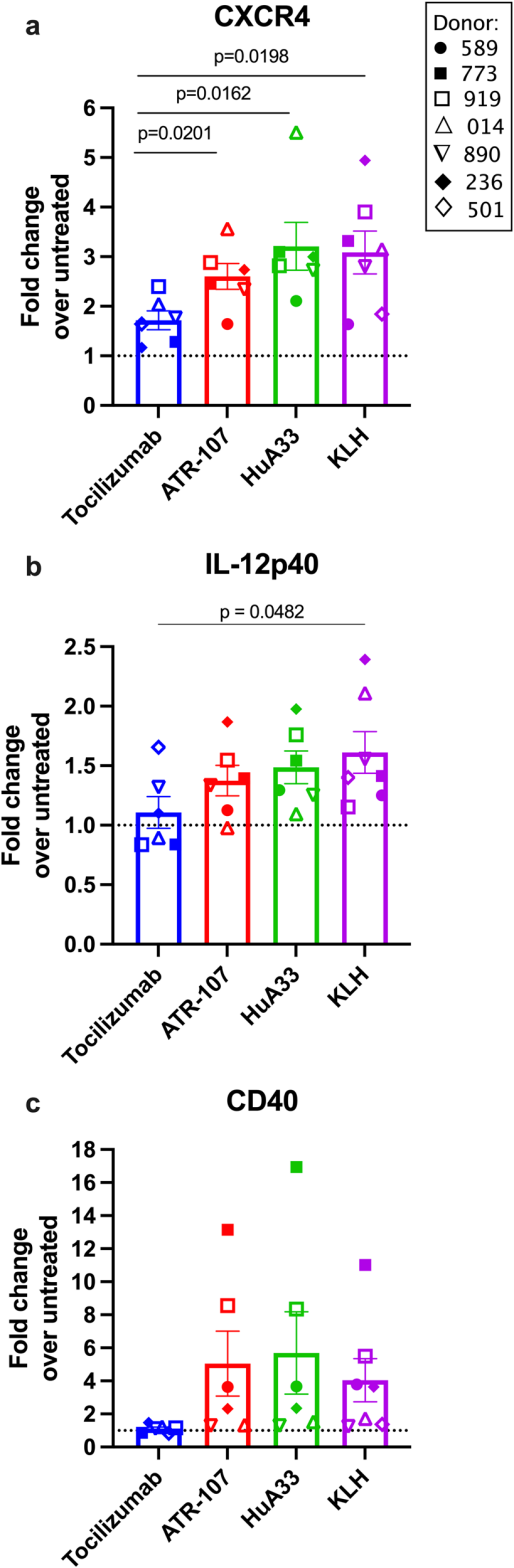
CXCR4 is upregulated on dendritic cells with activation markers CD40 and IL-12 by therapeutic proteins in proportion to their immunogenic potential. MoDCs from healthy donors (*n* = 6–7, Supplementary Table 1) were treated overnight with 5 μg/mL ATR-107, HuA33, tocilizumab, or KLH, plus 100 ng/mL LPS. Results are presented as the fold-change over the untreated control (media alone) for **a** % CXCR4^+^ moDCs, **b** % IL-12p40^+^ moDCs, and **c** % CD40^high^ moDCs out of the total CD11c^+^ HLA-DR^+^ moDC population. The dashed line at fold-change = 1 indicates no response. Treatments were tested in triplicate (*n* = 3 wells) for each donor. Each dot represents the mean response of one donor: (● closed circle) 589, (■ closed square) 773, (□ open square) 919, (△ open triangle) 014, (∇ upside-down triangle) 890, (◆ closed diamond) 236, and (◊ open diamond) 501. All bars are mean ± SEM. The gating strategy is presented in Supplementary Fig. 1. Statistical significance was determined by the student’s unpaired *t*-test (two-tailed) (*α* = 0.05) between each group. Exact *p*-values are reported unless *p* > 0.05, i.e., not significant. Estimated effect sizes (Cohen’s *d*) are located in Supplementary Data.

To compare results between donors, the fold-change over untreated was calculated by dividing the frequency (%) of CXCR4^+^ moDC in each treatment group by the average % CXCR4^+^ moDC in the untreated group. For KLH, ATR-107, and HuA33 in a population of six donors, the mean fold-change over untreated was significantly higher compared to tocilizumab (*p* = 0.0198, 0.0201, and 0.0162, respectively) (Fig. 2a). We then sought to confirm that therapeutic proteins with immunogenic risk upregulate DC maturation markers concurrently with CXCR4. Upon activation and maturation, DCs upregulate expression of CD40, and engagement with CD40L on naive CD4^+^ T cells promotes IL-12 production by DCs and differentiation of IFNγ-producing T helper 1 (Th1) cells^47,48^. Here, changes in CD40 expression and IL-12 production by DCs in response to therapeutic protein were captured by the frequency of CD40^high^ and IL-12p40^+^ moDCs which was then converted to fold-change over the untreated control (Supplementary Fig. 1 and Fig. 2b, c).

In response to treatment with ATR-107 and HuA33, an increase in the frequency of IL-12-producing (IL-12p40^+^) moDCs was observed. The fold-change over untreated was 1.38 ± 0.13 for ATR-107 and 1.49 ± 0.14 for HuA33 in six healthy donors, which was comparable to KLH (1.61 ± 0.17) (mean ± SEM) (Fig. 2b and Table 1). Tocilizumab only slightly upregulated IL-12p40^+^ moDC over baseline, with an average fold-change of 1.11 ± 0.13, which was significantly less than KLH (*p* = 0.0482). The frequency of CD40^high^ moDCs was also upregulated by KLH, ATR-107, and HuA33, indicated by an average fold-change of 4.04 ± 1.3, 5.05 ± 2.0, and 5.69 ± 2.5, respectively (mean ± SEM) (Fig. 2c and Table 1). Thus, compared to tocilizumab (1.12 ± 0.10), the fold-change in CD40 expression was 4-times greater (ns) for therapeutic proteins with high immunogenic risk. Upregulation of migration (CXCR4) and activation (IL-12, CD40) markers on dendritic cells captured the greater intrinsic immunogenic potential of ATR-107 and HuA33 compared to tocilizumab.

**Table 1:** The clinically reported anti-drug antibody incidence for therapeutic proteins and in vitro screening tool readouts for immunogenicity risk in healthy human donors.

	CXCR4+ % (fold-change over untreated)	CD40 high % (fold-change over untreated)	IL-12p40+ % (fold-change over untreated)	Total response index	Positive responses (%)	ADA incidence (%)
Tocilizumab	1.72 (*d* = 6)	1.12 (*d* = 6)	1.11 (*d* = 6)	1.31 (*n* = 18)	5.6 (1/18)	0.8–2
Rituximab	2.03 (*d* = 6)	1.36 (*d* = 6)	1.22 (*d* = 6)	1.53 (*n* = 18)	16.7 (3/18)	2–12
Emicizumab	2.97 (*d* = 6)	1.46 (*d* = 6)	1.06 (*d* = 6)	1.83 (*n* = 18)	16.7 (3/18)	5.1
Trastuzumab	2.27 (*d* = 6)	1.42 (*d* = 6)	1.15 (*d* = 6)	1.62 (*n* = 18)	11.1 (2/18)	16
Adalimumab	1.31 (*d* = 6)	1.73 (*d* = 6)	1.03 (*d* = 6)	1.36 (*n* = 18)	5.6 (1/18)	3–28^a^
ATR-107	2.44 (*d* = 6)	5.05 (*d* = 6)	1.37 (*d* = 6)	2.95 (*n* = 18)	50 (9/18)	76
HuA33	3.04 (*d* = 6)	5.69 (*d* = 6)	1.49 (*d* = 6)	3.41 (*n* = 18)	50 (9/18)	73
KLH	3.09 (*d* = 7)	4.04 (*d* = 7)	1.61 (*d* = 7)	2.91 (*n* = 21)	48 (10/21)	100

The fold-change over untreated for each marker is the mean of responses in *d* donors, and the total response index is the mean of *n* responses in all donors. Percent positive responses was determined as the percent responses greater than the 15th percentile of the LPS control group (2.21) out of the total responses. Monoclonal antibody target, host structure, route of administration, and references for ADA values are found in Supplementary Table 2.

*ADA* anti-drug antibody.

^a^Adalimumab biosimilar trials have demonstrated comparable ADA incidence for the biosimilar and reference product ranging from 32.0% up to 80.4%^69^.

### The combined DC marker readout correlates with immunogenicity incidence for a panel of therapeutic proteins

**Fig. 3:**
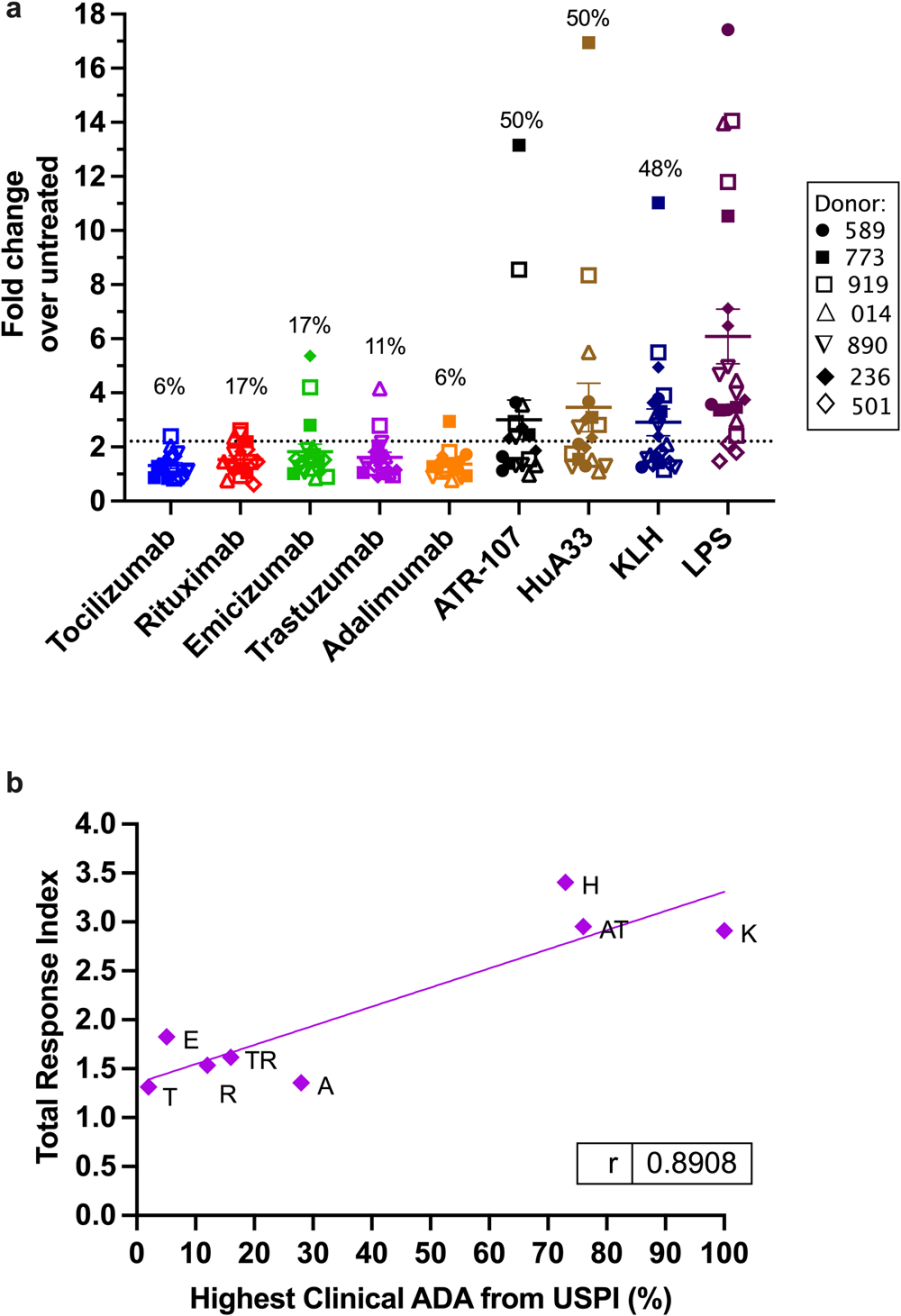
Correlations between the fold-change in predictive moDC markers for therapeutic proteins and their clinical anti-drug antibody incidence. **a** Fold-change over untreated for each DC marker (CXCR4^+^, CD40^high^, and IL-12p40^+^ % of moDC). Each dot represents the mean response of one donor for one marker. Each protein was tested in 6 to 7 donors for *n* = 18–21 total data points. Donors are indicated as: (● closed circle) 589, (■ closed square) 773, (□ open square) 919, (△ open triangle) 014, (∇ upside-down triangle) 890, (◆ closed diamond) 236, and (◊ open diamond) 501. Bars are mean ± SEM. Above each data set is the percentage of positive responses. Positive responses were those greater than the 15th percentile of the LPS control group (2.21). Donor information is available in Supplementary Table 1. **b** The “total response index” (mean fold-change in all markers for all donors (*n* = 6–7)) for each therapeutic protein versus the highest reported clinical ADA incidence from the USPI. Dots are labeled as: (T) tocilizumab, (E) emicizumab, (R) rituximab, (TR) trastuzumab, (A) adalimumab, (H) HuA33, (AT) ATR-107, and (K) KLH. The correlation coefficient (Pearson r) is shown. Sources for ADA incidence are available in Supplementary Table 2. *ADA* anti-drug antibody, *USPI* United States package insert.

The panel of protein antigens was expanded to include adalimumab, trastuzumab, rituximab, and emicizumab. Clinical immunogenicity incidence upon SC administration is available for all of these antibodies (Table 1 and Supplementary Table 2)^20,25,44–46,49^. MoDC from six to seven donors (Supplementary Table 1) were exposed to therapeutic protein as in the previous section. For each donor, the frequency of CXCR4^+^, IL-12p40^+^, and CD40^high^ moDC in the response to therapeutic protein was converted to fold-change over untreated moDC. Then for each marker, the mean values of each donor were pooled and correlated with ADA incidence to obtain Pearson r coefficients (Supplementary Fig. 2). All markers correlated positively with the highest clinical ADA incidence from US package inserts, with r values of 0.55, 0.86, and 0.92 for CXCR4, CD40, and IL-12p40, respectively. The results for all three markers in all donors were plotted together and the mean for each therapeutic protein was designated the “total response index” (Fig. 3a and Table 1). To determine the change in marker expression that was considered a “positive response”, we calculated the 15th percentile of the data set containing all marker expression levels for “mature DC” treated with 1 μg/mL LPS overnight. Any fold-change above 2.21 was considered a positive response, and the percent positive responses out of the total responses (# of donors x 3 markers) were calculated for each therapeutic protein (Fig. 3a and Table 1).

The total response index for each therapeutic protein was plotted against the highest clinical ADA incidence from US package inserts (Fig. 3b). A strong positive correlation was observed with a Pearson r coefficient equal to 0.87. The total response index for highly immunogenic proteins, KLH, ATR-107, and HuA33, was in the range of 2.9 to 3.5, while a range of 1.4 to 1.8 was observed for proteins with low to moderate immunogenicity incidence, namely, adalimumab, rituximab, trastuzumab, and emicizumab (Fig. 3b and Table 1). Then the monoclonal antibody with the lowest clinical immunogenicity incidence, tocilizumab, had a total response index of 1.3.

### Immunogenic therapeutic proteins not only upregulate CXCR4 but stimulate dendritic cell migration in a transwell assay

**Fig. 4:**
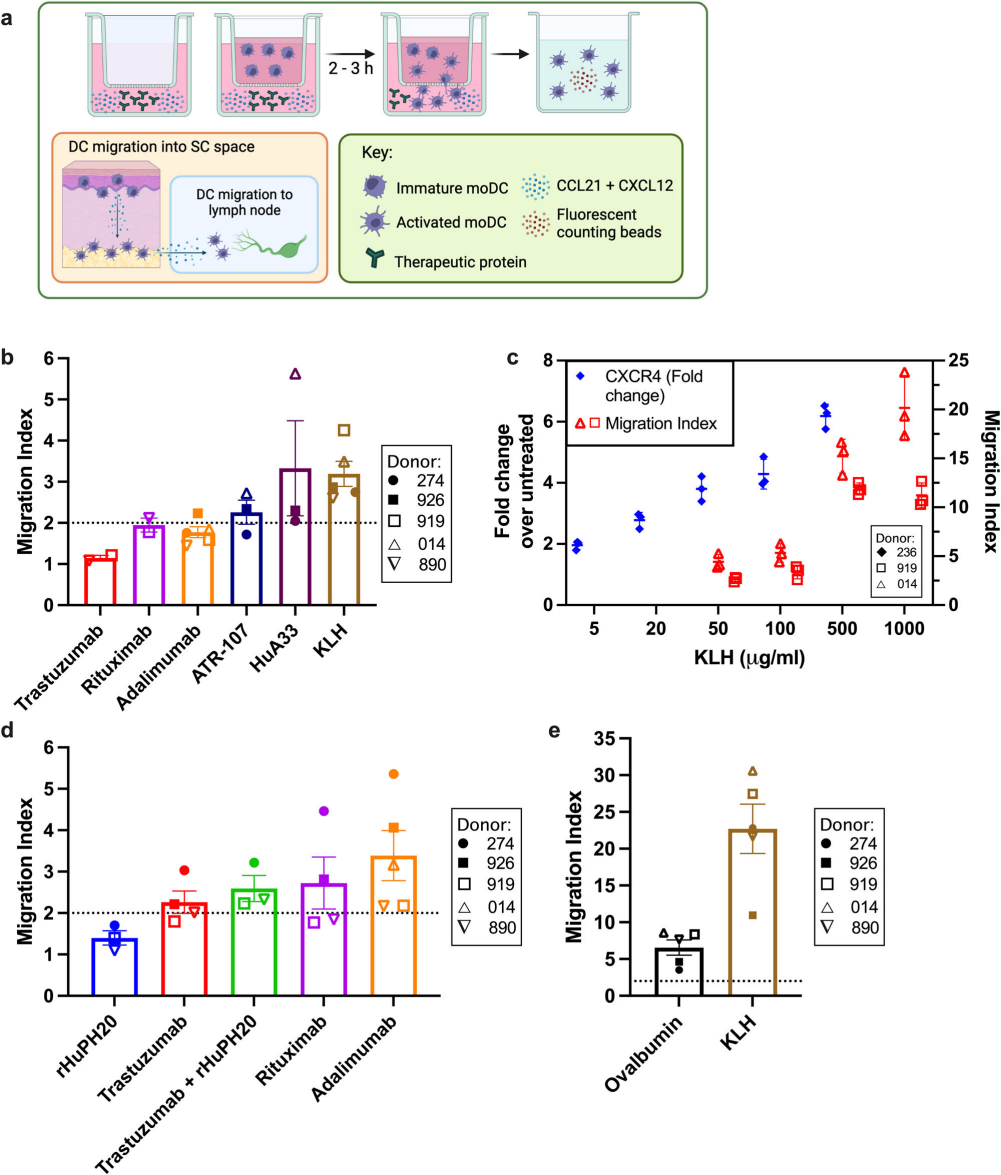
Migration of moDC toward CCL21 and CXCL12 in the presence of therapeutic protein correlates with its immunogenic potential. **a** Schematic of the transwell migration assay capturing the potential for DC migration toward therapeutic protein in the SC space. A concentration gradient of therapeutic protein and chemokines was created across the transwell insert. Immature DCs were plated in the upper chamber and allowed to migrate into the lower chamber. Migrated DCs were counted in the lower chamber by flow cytometry. Schematic created at Biorender.com. **b** The migration index of moDC along a concentration gradient of therapeutic protein in the presence of CCL21 and CXCL12. Cells were plated in the upper chambers of transwell inserts with 10 μg/mL therapeutic protein. 50 μg/mL therapeutic protein plus 100 ng/mL each CCL21 and CXCL12 were added to lower chambers. Migration index = (% migrated of protein treatment group)/(average % migrated of “media” control), where “media” has only chemokines in the lower chamber. **c** (*Left y-axis*) The fold-change in CXCR4^+^ (%) moDC over untreated as a function of KLH concentration for a representative donor: (◆ closed diamond) 236. (*Right y-axis*) The migration index of moDC as a function of KLH concentration in the lower chamber for representative donors: (□ open square) 919, (△ open triangle) 014. Each dot is a technical replicate (*n* = 3 wells); error bars are mean ± SD. **d**, **e** Cells were plated in the upper chambers of transwell inserts with 100 μg/mL therapeutic protein. 1000 μg/mL therapeutic protein plus 100 ng/mL each CCL21 and CXCL12 were added to lower chambers. In **b**, **d**, **e** treatments were tested in triplicate (*n* = 3 wells) for each donor, and each dot represents the mean for one donor: (● closed circle) 274, (■ closed square) 926, (□ open square) 919, (△ open triangle) 014, (∇ upside-down triangle) 890, (◆ closed diamond) 236. Error bars are mean ± SEM.

We hypothesize that immunogenic risk is increased when signals from the immunogenic potential of the protein and any danger signals in the formulation combine to increase dendritic cell migration into and out of the injection site^18^. In these experiments, we sought to capture the migration potential of DCs toward therapeutic protein in vitro using a transwell assay. The assay was set up to test whether immunogenic proteins drive DC migration in the presence of chemokine ligands for receptors CCR7 and CXCR4, which are CCL21 and CXCL12, respectively (Fig. 4a). In preliminary experiments, DC migration toward a combined gradient of CCL21 + CXCL12—providing receptor-ligand interactions for CCR7 and CXCR4—was stronger than migration toward only CCR7 ligands (CCL21 + CCL19) (Supplementary Fig. 3a). In the final transwell method, the bottom chambers of the transwell insert contained 100 ng/mL each CCL21 and CXCL12 plus therapeutic protein, and immature moDC were added to the upper chambers with therapeutic protein. First, highly immunogenic proteins were screened in the transwell test with a low concentration gradient of (top) 10 μg/mL to (bottom) 50 μg/mL protein with CCL21 + CXCL12 in the bottom chamber. In this condition, KLH and HuA33 induced 3-fold more moDC migration into the bottom chamber compared to when only chemokine ligands were present (Fig. 4b). The fold-change in migration is referred to as the “migration index”. We found the migration index to be dependent on the concentration of protein added to the lower chamber while keeping the concentration of CCL21 and CXCL12 constant (Fig. 4c and Supplementary Fig. 3b). The migration index also correlated with the fold-change in CXCR4^+^ moDC (Fig. 4c).

For monoclonal antibodies with low to moderate immunogenic risk (rituximab, trastuzumab, and adalimumab), the migration index was similar when tested at the concentration gradient 10 μg/mL to 50 μg/mL protein (Fig. 4b). Thus, in order to screen low to moderate immunogenic risk, and since the migration index was found to be concentration dependent, the concentration gradient was increased to (top) 100 μg/mL and (bottom) 1000 μg/mL protein. The concentrations of CCL21 and CXCL12 in the bottom chamber were kept constant (100 ng/mL each). Compared to rituximab and trastuzumab, adalimumab induced the greatest migration index (3.4 ± 0.6) in five donors (mean ± SEM) (Fig. 4d). In the same donors, the mean migration index was 2.7 ± 0.6 for rituximab and 2.3 ± 0.3 for trastuzumab, while KLH induced an average migration index of 23 ± 3 (Fig. 4e). We were unable to screen ATR-107 and HuA33 at the high concentration gradient, but migration index results at the 10 to 50 μg/mL condition suggest moDC would migrate robustly in response to these proteins (e.g., migration index > 3.5).

## Discussion

Unique immunogenicity challenges are introduced by the subcutaneous route of administration for biologics compared to other delivery routes^17^. It is imperative to perform immunogenicity risk assessment during development stages, but predictive power and mechanistic insight are lacking for many available methods. Here the migratory potential of dendritic cells was transformed into a marker for immunogenic risk since dendritic cell migration is a driving step of T-cell-dependent immune responses^17,18^. Migratory potential was captured by expression of CXCR4 and combined with activation markers CD40 and IL-12 to generate the readout of the “total response index”. CXCR4 has not been considered as a marker for immunogenic risk; however, we found it to be reliably upregulated in proportion to a protein’s immunogenic potential (Figs. 1 and 2a). Then in an additional testing system, DC migration in the presence of therapeutic protein was captured by a transwell assay where chemokine ligands for CXCR4 and CCR7 were included (CXCL12 and CCL21, respectively). Upregulation of CXCR4 on moDCs corresponded to an increase in DC migration toward the combination of chemokine ligands (Fig. 4c).

In the first testing system, the total response index captured the responses of healthy donors (n = 6–7) toward therapeutic proteins as the fold-change in % CXCR4^+^, IL-12p40^+^, and CD40^high^ moDCs over untreated (Figs. 2 and  3a). DCs expressing CXCR4 and CD40 in addition to producing IL-12 will be strong inducers of CD4^+^ T-cell activation and Th1 cell differentiation^31,47^. For ATR-107 and HuA33, the fold-change in each marker was similar in value to the positive control KLH, which is a protein with very high immunogenic potential (Fig. 2). In retrospective immunogenicity assessment of ATR-107, Xue and colleagues at Pfizer found that it induced expression of DC maturation markers, such as CD86 and CD40, substantially more than a control antibody^50^. Furthermore, they found a population of CCR7-expressing DCs that was upregulated in the presence of ATR-107, which corresponds to our findings that ATR-107 can induce moDC migration toward CCL21 in the transwell assay (Fig. 4b). Similar outcomes were observed for the immunogenic mAb HuA33. For example, the mean migration index was 5.6 in a donor (#014) who displayed strong upregulation of CXCR4 in the presence of HuA33 (fold-change = 5.5) (Figs. 2a and 4b). Even though HuA33 and ATR-107 differ greatly in target and humanization (Supplementary Table 2), the total response index predicted their high immunogenic potential (Fig. 3b).

The total response index was found to correlate positively with the immunogenic potential of the therapeutic proteins tested (*r* = 0.87) (Fig. 3b). Immunogenicity potential was represented by the highest reported clinical ADA incidence since many proteins show high variability in ADA incidence between studies^51^. Predicting the risk of immunogenic response, by determining the severity of the event and/or the probability of occurrence, is the goal for an in vitro screening tool. Here we used the total response index as an output for the severity of risk, and we also reported the number of positive responses out of the total responses obtained (# of donors x 3 markers) (Table 1). When testing each pool of donor cells, moDC were treated with 1 μg/mL LPS to induce full maturation and upregulate all markers (CD40, CXCR4, and IL-12). The mean of all marker responses for LPS was 6.1 (*n* = 7 donors) and the 15^th^ percentile of this data (2.21) was used to set the positive response threshold to preliminarily determine the number of positive responses. For ATR-107 and HuA33, having high immunogenic potential, the percent of positive responses was 50% (*n* = 9/18) (Table 1 and Fig. 3a). For tocilizumab, only 6% (1/18) positive responses were observed, and rituximab and emicizumab showed a 16.6% positive response rate (*n* = 3/18) (Table 1 and Fig. 3a). The total response index better captured the difference in immunogenic potential between rituximab and trastuzumab compared to the positive response rate (Table 1 and Fig. 3a).

Adalimumab showed only 6% positive responses (*n* = 1/18) and a comparatively low total response index (1.36) which does not reflect its greater immunogenicity incidence in comparison to trastuzumab, rituximab, emicizumab, or tocilizumab (Table 1). The immunogenic potential of adalimumab can be difficult to capture by in vitro assessment tools^8,52^. The mechanism of action of tumor necrosis factor (TNF)-inhibitors has interfered with immunogenicity risk assessment based on T-cell activation, such as T helper cell expression of CD134/CD137^52^. TNF blocking in vitro can delay CD4^+^ T-cell activation and proliferation, and reduced T-cell activation has been observed in inflammatory bowel disease populations treated with TNF inhibitors^53^. Delay of T-cell activation by the TNF inhibitor does not rule out future immunogenic response toward the biotherapeutic since there were limited effects on the long-term viability of T cells and their ability to respond to stimulation^53^. Our data indicate that the total response index did not accurately predict the relative immunogenicity of adalimumab due to the impact of TNF blocking on DC maturation and cytokine production^54^. However, the transwell assay demonstrated better predictive power for adalimumab by predicting a higher risk of immunogenicity than trastuzumab or rituximab, based on the migration index (Fig. 4d). Results suggest that the transwell assay may be less susceptible to interference by the biotherapeutic’s mechanism of action.

The possibility of receptor-mediated interactions between DCs and therapeutic proteins, either via the target antigen or other surface receptors, should be considered when using DC-based immunogenicity screening assays. For example, the interaction of proteins with DC surface receptors used for antigen uptake (e.g., mannose receptor or DEC-205), could increase the possibility of immunogenic risk by enhanced antigen processing and presentation^55–57^. Additionally, expression of the target antigen by DCs could promote the uptake of the protein and directly provide stimulatory or modulatory signals to the DCs. For example, blocking of the IL-6/IL-6R signaling pathway by tocilizumab is expected to contribute to its low incidence of immunogenicity^58^. Direct interaction of tocilizumab with the IL-6 receptor expressed by DCs^59^ likely contributed to the low immunogenic potential predicted by our in vitro screening assay. This circumstance would not apply to proteins whose targets have little to no expression on DCs, such as trastuzumab (anti-HER2) and rituximab (anti-CD20). Although the implications of DC binding on immunogenic risk are complicated. For example, DC binding was not predictive of immunogenic risk for ATR-107 which has a therapeutic target expressed by DCs (IL-21R). The magnitude of DC binding by ATR-107 was not meaningfully different than a control antibody with low immunogenic potential (PF-1)^50^. As for HuA33, other factors besides the therapeutic target (glycoprotein A33) are expected to contribute to its high immunogenic potential since GPA33 has little expression on human DCs and monocytes^60^.

Recommended use of the total response index and transwell migration index in an immunogenicity screening toolbox for biotherapeutics is demonstrated in Supplementary Fig. 4. These in vitro testing systems for immunogenicity risk assessment are well aligned with FDA recommendations in the Modernization Act 2.0 for the use of human biology-based “nonclinical” tests with less reliance on preclinical animal-based approaches^61^. Future expansion of the donor population screened by the assays in our toolbox, for example, at least 10 HLA-diverse donors, would allow for the determination of sensitivity and false positive rates, beyond the preliminary descriptive statistical analysis performed here^39,43^.

By capturing the likelihood of DC migration from the epidermis/dermis to the hypodermis, we can use the transwell assay to test for the impact of therapeutic protein characteristics on immunogenic risk following SC administration. We found DC migration to be concentration dependent, with concentrations of 1 mg/mL KLH inducing strong migration into the bottom transwell chamber (i.e., 30–50% migrated) (Supplementary Fig. 3b). Injection site concentrations of mAb are likely sufficient to induce strong DC migration from the upper layers of skin, according to the immunogenic potential of the mAb, since high concentrations of protein (e.g., 100 mg/mL) are typically formulated for SC administration. Product characteristics can also impact the likelihood of DC migration, such as protein structural features, instability pathways (e.g., aggregates), or formulation properties (e.g., viscosity) that prolong injection site retention time^17,19^. Research-grade biotherapeutics, as used here, may differ in aggregation levels compared to clinical preparations, which is a confounding factor in extrapolating our in vitro results to clinical immunogenicity. Aggregation potential can be determined using the folding model, and determination of aggregate levels in products tested for in vitro immunogenicity risk would strengthen confidence in extrapolation to immunogenicity of clinical preparations^62^.

Other inherent product characteristics could contribute to immunogenicity, such as the ability of some mAbs to form large complexes with soluble target^63^. Our screening assay could incorporate the testing of such product characteristics, for example, by exposing dendritic cells to the drug in the presence of a soluble target. Many other product-related risk factors could be screened in the transwell migration assay, and the ability of the assay to predict the impact of stressed protein molecules on moDC migration is currently under investigation. In an in vitro skin model, aggregated mAbs were found to induce proinflammatory cytokine and chemokine production by skin cells, suggesting a mechanism by which aggregates could increase DC migration into the SC injection site^64^. The injection of hyaluronidase with highly concentrated mAbs can improve absorption time and bioavailability; however, the immunogenicity of the co-administered mAb is not reduced^20^. Furthermore, we found that hyaluronidase increased the migration of moDC toward trastuzumab for two donors, although a statistically significant difference was not achieved (Fig. 4d).

We strongly believe the applicability of this in vitro testing system for immunogenicity extends beyond proteins and toward novel biological modalities, in addition to recognizing risk introduced by aggregation, changes in formulation, protein structure/post-translational modifications, concentration, and more. Novel therapeutic modalities, like AAV vectors, CAR T cells, nucleic acids, and so on, have unique immunogenicity concerns and appear to differ in immune activation mechanisms compared to the therapeutic proteins^65–68^. However, since dendritic cells are extensively involved in immune responses toward all types of antigens, they remain a feasible cellular option for in vitro screening of novel modalities. Introducing mechanism-based markers into immunogenicity risk assessment of biologics should improve predictive power and the understanding of risk factors behind immunogenicity.

### Supplementary information

Supplementary Information

Description of Additional Supplementary Files

Supplementary Data

Reporting Summary

## Data Availability

All data are contained within the manuscript text, Supplementary Information, and Supplementary Data. Source data for Figs. 1–4 and Supplementary Figs. 2 and 3 can be found in the Supplementary Data file.

## References

[1] Wincup C (2023). Anti-rituximab antibodies demonstrate neutralizing capacity, associate with lower circulating drug levels and earlier relapse in lupus. Rheumatology (Oxford).

[2] Linthorst GE, Hollak CE, Donker-Koopman WE, Strijland A, Aerts JM (2004). Enzyme therapy for Fabry disease: neutralizing antibodies toward agalsidase alpha and beta. Kidney Int.

[3] Ljung R (2019). Inhibitors in haemophilia A and B: management of bleeds, inhibitor eradication and strategies for difficult-to-treat patients. Eur. J. Haematol..

[4] Banugaria SG (2012). Persistence of high sustained antibodies to enzyme replacement therapy despite extensive immunomodulatory therapy in an infant with Pompe disease: need for agents to target antibody-secreting plasma cells. Mol. Genet. Metab..

[5] Casadevall N (2002). Pure red-cell aplasia and antierythropoietin antibodies in patients treated with recombinant erythropoietin. N. Engl. J. Med..

[6] Ridker PM (2017). Lipid-reduction variability and antidrug-antibody formation with bococizumab. N. Engl. J. Med..

[7] Mullin, R. Pfizer discontinues work on bococizumab. *Chem. Eng. News* **94**, ISSN 0009-2347 (2016).

[8] Kraus T (2019). Evaluation of a 3D human artificial lymph node as test model for the assessment of immunogenicity of protein aggregates. J. Pharmaceut. Sci..

[9] Lycke N, Coico R (2015). ELISPOT assay for measurement of antigen-specific and polyclonal antibody responses. Curr. Protoc. Immunol..

[10] Czerkinsky CC, Nilsson LA, Nygren H, Ouchterlony O, Tarkowski A (1983). A solid-phase enzyme-linked immunospot (ELISPOT) assay for enumeration of specific antibody-secreting cells. J. Immunol. Methods.

[11] Jawa, V. et al. T-cell dependent immunogenicity of protein therapeutics pre-clinical assessment and mitigation–updated consensus and review 2020. *Front. Immunol.* **11**, 10.3389/fimmu.2020.01301 (2020). 10.3389/fimmu.2020.01301 PMC7338774 32695107

[12] Karle AC (2020). Applying MAPPs assays to assess drug immunogenicity. Front. Immunol..

[13] Gokemeijer J, Jawa V, Mitra-Kaushik S (2017). How close are we to profiling immunogenicity risk using in silico algorithms and in vitro methods?: an industry perspective. AAPS J.

[14] Brinks V (2013). Preclinical models used for immunogenicity prediction of therapeutic proteins. Pharm. Res..

[15] Alvarez D, Vollmann EH, von Andrian UH (2008). Mechanisms and consequences of dendritic cell migration. Immunity.

[16] Förster R, Braun A, Worbs T (2012). Lymph node homing of T cells and dendritic cells via afferent lymphatics. Trends Immunol..

[17] Jarvi NL, Balu-Iyer SV (2021). Immunogenicity challenges associated with subcutaneous delivery of therapeutic proteins. BioDrugs.

[18] Fathallah AM, Bankert RB, Balu-Iyer SV (2013). Immunogenicity of subcutaneously administered therapeutic proteins–a mechanistic perspective. AAPS J..

[19] Hamuro L (2017). Perspectives on subcutaneous route of administration as an immunogenicity risk factor for therapeutic proteins. J. Pharm. Sci..

[20] Herceptin Hylecta [package insert]. South San Francisco, CA (Genentech, Inc., 2019) (revised). https://www.accessdata.fda.gov/drugsatfda_docs/label/2019/761106s000lbl.pdf Accessed 7 Oct 2020.

[21] Ogata A (2014). Phase III study of the efficacy and safety of subcutaneous versus intravenous tocilizumab monotherapy in patients with rheumatoid arthritis. Arthritis Care Res. (Hoboken).

[22] Omontys [package insert]. Deerfield, IL (Takeda Pharmaceuticals America, Inc., 2012) (revised). https://www.accessdata.fda.gov/drugsatfda_docs/label/2012/202799s001lbl.pdf Accessed 10 Oct 2020.

[23] Ortega HG (2014). Mepolizumab treatment in patients with severe eosinophilic asthma. N. Engl. J. Med..

[24] Phesgo [package insert]. South San Francisco, CA (Genentech, Inc., 2020 (revised)). https://www.accessdata.fda.gov/drugsatfda_docs/label/2020/761170s000lbl.pdf Accessed 7 Oct 2020.

[25] Rituxan Hycela [package insert]. South San Francisco, CA (Genentech, Inc., 2020) (revised). https://www.accessdata.fda.gov/drugsatfda_docs/label/2020/761064s008s010lbl.pdf Accessed 7 Oct 2020.

[26] Zhuang Y (2012). Golimumab pharmacokinetics after repeated subcutaneous and intravenous administrations in patients with rheumatoid arthritis and the effect of concomitant methotrexate: an open-label, randomized study. Clin. Ther..

[27] Itano AA (2003). Distinct dendritic cell populations sequentially present antigen to CD4 T cells and stimulate different aspects of cell-mediated immunity. Immunity.

[28] Worbs T, Hammerschmidt SI, Förster R (2017). Dendritic cell migration in health and disease. Nat. Rev. Immunol..

[29] Levin C (2017). Critical role for skin-derived migratory DCs and langerhans cells in TFH and GC responses after intradermal immunization. J. Investig. Dermatol..

[30] Comerford I (2013). A myriad of functions and complex regulation of the CCR7/CCL19/CCL21 chemokine axis in the adaptive immune system. Cytokine Growth Factor Rev..

[31] Kabashima K (2007). CXCL12-CXCR4 engagement is required for migration of cutaneous dendritic cells. Am. J. Pathol..

[32] Förster R, Davalos-Misslitz AC, Rot A (2008). CCR7 and its ligands: balancing immunity and tolerance. Nat. Rev. Immunol..

[33] Ohl L (2004). CCR7 governs skin dendritic cell migration under inflammatory and steady-state conditions. Immunity.

[34] Dalod M, Chelbi R, Malissen B, Lawrence T (2014). Dendritic cell maturation: functional specialization through signaling specificity and transcriptional programming. Embo J..

[35] Nace G, Evankovich J, Eid R, Tsung A (2012). Dendritic cells and damage-associated molecular patterns: endogenous danger signals linking innate and adaptive immunity. J. Innate Immun..

[36] Hiasa M (2009). GM-CSF and IL-4 induce dendritic cell differentiation and disrupt osteoclastogenesis through M-CSF receptor shedding by up-regulation of TNF-α converting enzyme (TACE). Blood.

[37] Posch, W., Lass-Flörl, C. & Wilflingseder, D. Generation of human monocyte-derived dendritic cells from whole blood. *J. Vis. Exp.*10.3791/54968 (2016). 10.3791/54968 PMC5226452 28060313

[38] Grassi F (1998). Monocyte-derived dendritic cells have a phenotype comparable to that of dermal dendritic cells and display ultrastructural granules distinct from Birbeck granules. J. Leukoc. Biol..

[39] Walsh RE (2020). Post-hoc assessment of the immunogenicity of three antibodies reveals distinct immune stimulatory mechanisms. mAbs.

[40] Wen Y (2020). Development of a FRET-based assay for analysis of mAbs internalization and processing by dendritic cells in preclinical immunogenicity risk assessment. AAPS J..

[41] Wen Y (2021). Comparability study of monocyte derived dendritic cells, primary monocytes, and THP1 cells for innate immune responses. J. Immunol. Methods.

[42] Ducret A (2022). Assay format diversity in pre-clinical immunogenicity risk assessment: Toward a possible harmonization of antigenicity assays. MAbs.

[43] Wickramarachchi D (2020). Fit-for-purpose validation and establishment of assay acceptance and reporting criteria of dendritic cell activation assay contributing to the assessment of immunogenicity risk. AAPS J..

[44] Hua F (2014). Anti-IL21 receptor monoclonal antibody (ATR-107): Safety, pharmacokinetics, and pharmacodynamic evaluation in healthy volunteers: A phase I, first-in-human study. J. Clin. Pharmacol..

[45] Welt S (2003). Phase I study of anticolon cancer humanized antibody A33. Clin. Cancer Res..

[46] Genentech, Inc. https://www.accessdata.fda.gov/drugsatfda_docs/label/2020/125276s129,125472s042lbl.pdf. Actemra [package insert]. South San Francisco, CA: (Genentech, Inc., 2020 (revised)) Accessed 7 Oct 2020.

[47] Ma DY, Clark EA (2009). The role of CD40 and CD154/CD40L in dendritic cells. Semin. Immunol..

[48] Reinhardt RL, Hong S, Kang S-J, Wang Z-E, Locksley RM (2006). Visualization of IL-12/23p40 in vivo reveals immunostimulatory dendritic cell migrants that promote Th1 differentiation1. J. Immunol..

[49] Bartelds GM (2011). Development of antidrug antibodies against adalimumab and association with disease activity and treatment failure during long-term follow-up. JAMA.

[50] Xue L, Hickling T, Song R, Nowak J, Rup B (2015). Contribution of enhanced engagement of antigen presentation machinery to the clinical immunogenicity of a human interleukin (IL)-21 receptor-blocking therapeutic antibody. Clin. Exp. Immunol..

[51] Dingman R, Balu-Iyer SV (2019). Immunogenicity of protein pharmaceuticals. J. Pharm. Sci..

[52] Cohen S (2021). Immunogenicity risk assessment for biotherapeutics through in vitro detection of CD134 and CD137 on T helper cells. MAbs.

[53] Povoleri GAM (2020). Anti-TNF treatment negatively regulates human CD4( + ) T-cell activation and maturation in vitro, but does not confer an anergic or suppressive phenotype. Eur. J. Immunol..

[54] Helen MB, Toshiko I-I, John DI, Catharien MUH (2010). Tumour necrosis factor alpha blockade impairs dendritic cell survival and function in rheumatoid arthritis. Ann. Rheumat. Dis..

[55] Platt CD (2010). Mature dendritic cells use endocytic receptors to capture and present antigens. Proc. Natl Acad. Sci. USA.

[56] Mahnke K (2000). The dendritic cell receptor for endocytosis, DEC-205, can recycle and enhance antigen presentation via major histocompatibility complex class II-positive lysosomal compartments. J. Cell. Biol..

[57] Figdor CG, van Kooyk Y, Adema GJ (2002). C-type lectin receptors on dendritic cells and langerhans cells. Nat. Rev. Immunol..

[58] Gerd RB (2017). Low immunogenicity of tocilizumab in patients with rheumatoid arthritis. Ann. Rheumat. Dis..

[59] Verboogen DRJ, Revelo NH, ter Beest M, van den Bogaart G (2019). Interleukin-6 secretion is limited by self-signaling in endosomes. J. Mol. Cell Biol..

[60] Opstelten R (2021). GPA33 is expressed on multiple human blood cell types and distinguishes CD4(+) central memory T cells with and without effector function. Eur. J. Immunol..

[61] Adashi EY, O’Mahony DP, Cohen IG (2023). The FDA modernization Act 2.0: drug testing in animals is rendered optional. Am. J. Med..

[62] Rao G (2010). Use of a folding model and in situ spectroscopic techniques for rational formulation development and stability testing of monoclonal antibody therapeutics. J. Pharm. Sci..

[63] Kohno T, Tam L-TT, Stevens SR, Louie JS (2007). Binding characteristics of tumor necrosis factor receptor-fc fusion proteins vs anti-tumor necrosis factor mAbs. J. Investig. Dermatol. Symp. Proc..

[64] Tokuda, J. M. et al. Use of in vitro human skin models to assess potential immune activation in response to biotherapeutic attributes and process-related impurities. *J. Pharmaceut. Sci.*10.1016/j.xphs.2022.02.001 (2022). 10.1016/j.xphs.2022.02.001 35139332

[65] Verdera HC, Kuranda K, Mingozzi F (2020). AAV vector immunogenicity in humans: a long journey to successful gene transfer. Mol. Ther..

[66] Yang T-Y (2022). Immunogenicity assessment of AAV-based gene therapies: an IQ consortium industry white paper. Mol. Ther. Method. Clin. Dev..

[67] Stebbins CC, Petrillo M, Stevenson LF (2019). Immunogenicity for antisense oligonucleotides: a risk-based assessment. Bioanalysis.

[68] Gorovits B, Koren E (2019). Immunogenicity of chimeric antigen receptor T-cell therapeutics. BioDrugs.

[69] Lu X, Hu R, Peng L, Liu M, Sun Z (2021). Efficacy and safety of adalimumab biosimilars: current critical clinical data in rheumatoid arthritis. Front. Immunol.

