# Supplementary material for: A mechanistic marker-based screening tool to predict clinical immunogenicity of biologics
Source: Commun Med (Lond). 2023 Dec 8;3:174. doi: 10.1038/s43856-023-00413-7 (PMC10709359; doi:10.1038/s43856-023-00413-7)
Supplement: Supplementary file 1 — Supplementary Information [file 43856_2023_413_MOESM1_ESM.pdf]

Supplementary Figures and Figure Legends

Supplementary Figure 1. Flow cytometry gating strategy for human monocyte-derived dendritic cells

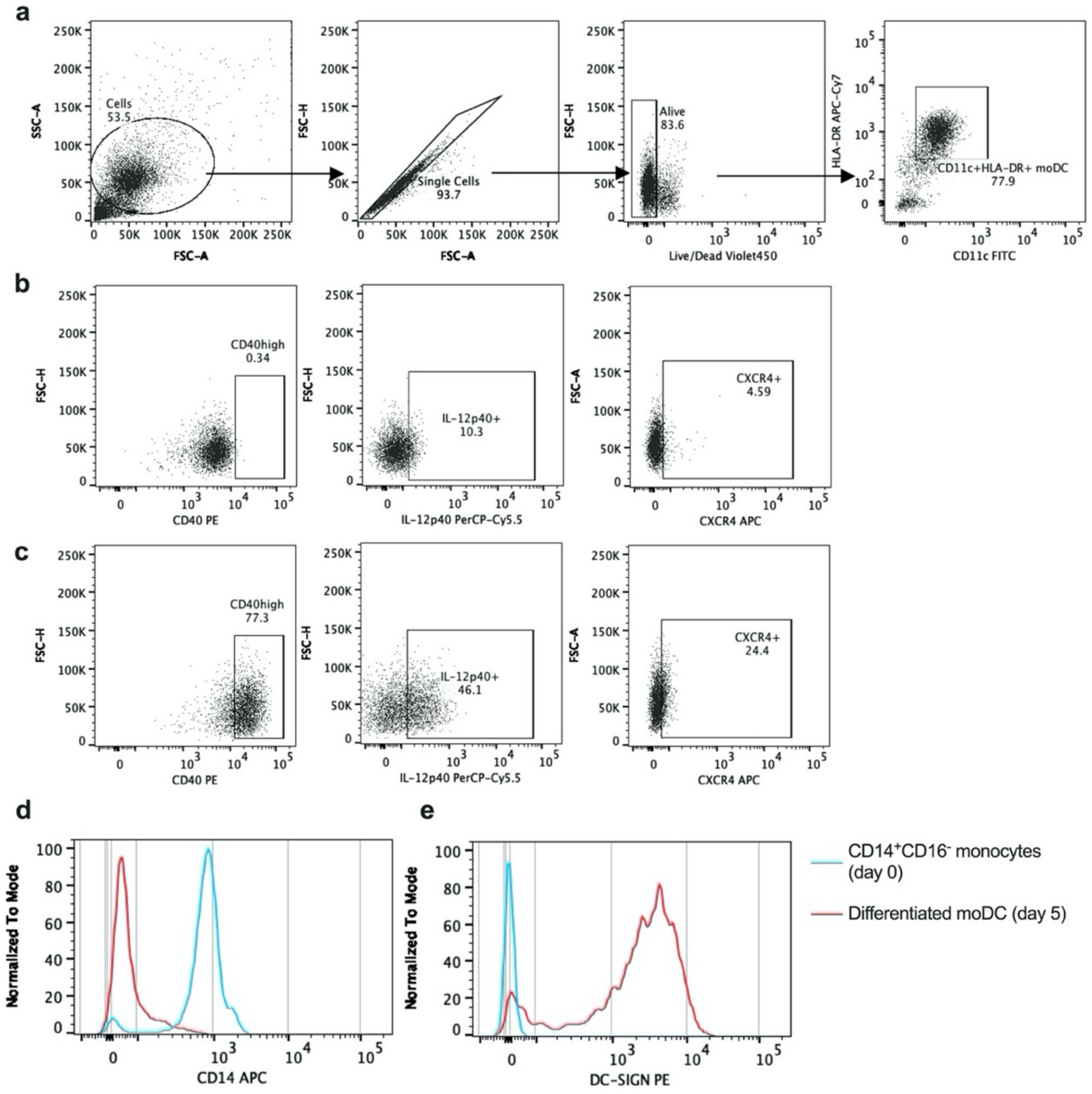

(a) Dot plots show a representative donor (#773) sample. Cells were gated based on FSC-A vs SSC-A then singlets were gated based on FSC-A vs FSC-H. Live cells were gated as Live/Dead $V450^{low}$ then moDC were gated as $CD11c^{+}HLA\text{-}DR^{+}$. **(b-c)** Gating strategy for $CD40^{high}$, $IL\text{-}12p40^{+}$, and $CXCR4^{+}$ moDCs (from left to right). MoDCs were treated overnight with **(b)** media or **(c)** 1 μg/mL LPS. **(d-e)** Representative histograms of **(d)** CD14 APC (monocyte marker) and **(e)** DC-SIGN PE (dendritic cell marker) on $CD14^{+}CD16^{-}$ monocytes pre-differentiation (culture day 0) and on differentiated moDCs (culture day 5), blue and red lines, respectively.

Supplementary Figure 2. Correlations between clinical anti-drug antibody incidence and the fold change over untreated for predictive moDC markers in response to therapeutic proteins

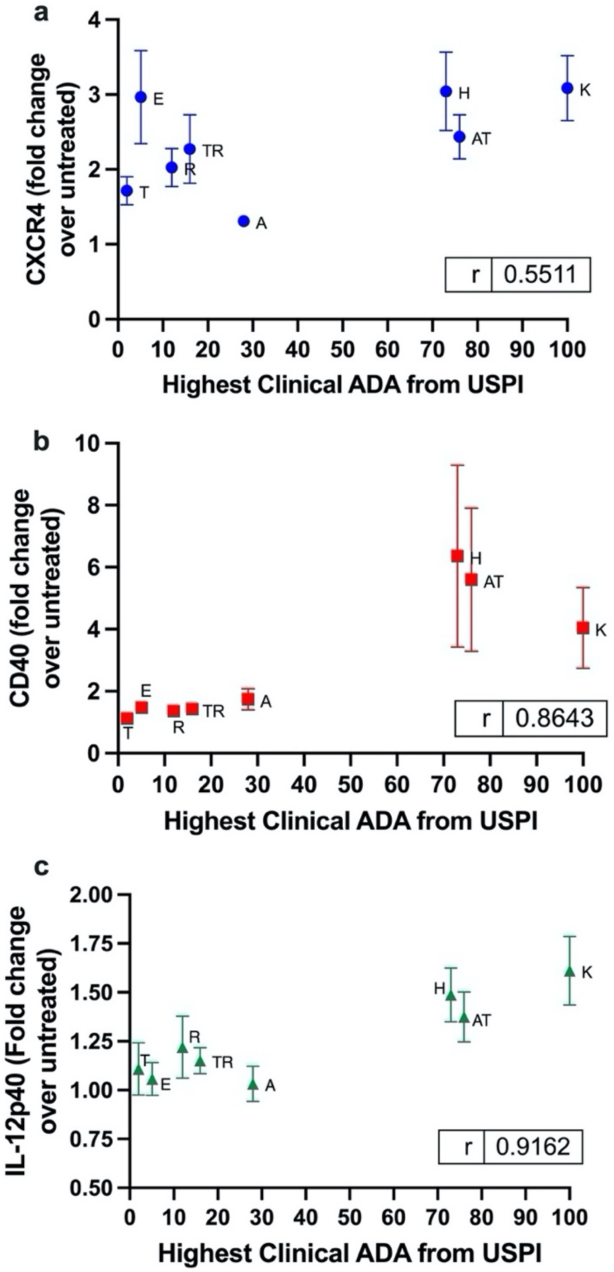

(a) Fold change in % CXCR4$^+$, **(b)** CD40^{high}, and **(c)** IL-12p40$^+$ moDCs over untreated versus clinical anti-drug antibody incidence. Each labeled dot represents the mean response of 6 to 7 donors toward therapeutic proteins: (T) tocilizumab, (E) emicizumab, (R) rituximab, (TR) trastuzumab, (A) adalimumab, (H) HuA33, (AT) ATR-107, and (K) KLH. All error bars are mean ± SEM. Sources for ADA incidence are found in Supp. Table 2. Correlation coefficients (Pearson r) are shown in each plot.

Supplementary Figure 3. Migration of DCs toward combined chemokine gradients in a Transwell assay

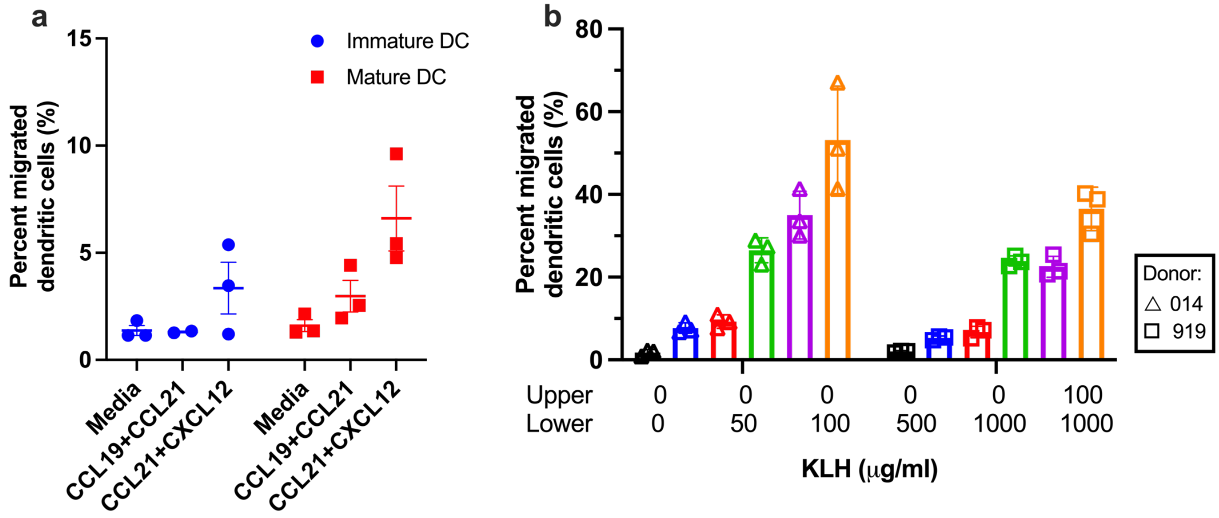

(a) The percent migrated dendritic cells (%) in the Transwell test for immature DC and mature DC (pre-treated overnight with media or 1 μg/mL LPS, respectively). Cells were plated in the upper chambers of a 96-well Transwell plate and lower chambers were filled with serum-free media containing no chemokines, CCL19 and CCL21, or CCL21 and CXCL12. All chemokines were plated at 200 ng/mL each. Treatments were tested in triplicate (n=3 wells). Bars are mean ± SEM. **(b)** Immature moDC from healthy human donors (n=2) were plated in the upper chambers of a 96-well Transwell plate. Lower chambers were filled with serum-free media containing 100 ng/mL CCL21 and 100 ng/mL CXCL12 with 0 to 1000 μg/mL KLH. In one group KLH was also added to the upper chamber (100 μg/mL). The graph shows the percent migrated DCs (%) under each condition. Donor 014 (△ open triangle). Donor 919 (□ open square) 919. Treatments were tested in triplicate (n=3 wells) for each donor; each dot represents a technical replicate. Bars are mean ± SEM.

Supplementary Figure 4. Proposed workflow of immunogenicity risk assessment using *in vitro* screening assays for dendritic cell migratory potential

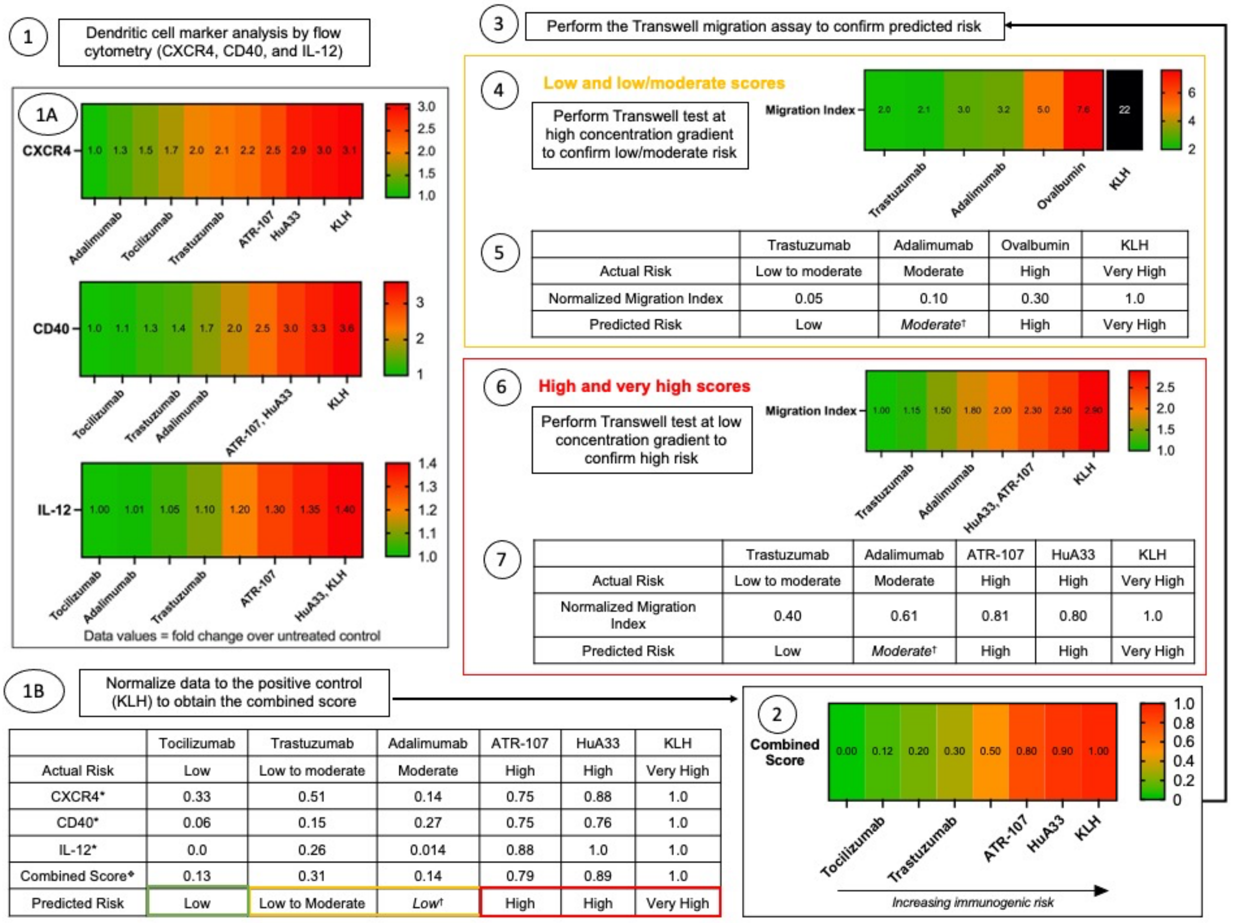

Step 1, Perform immunogenicity risk assessment via flow cytometry analysis of mechanism-driven markers on dendritic cells. Calculate fold change over untreated for $CXCR4^{+}$, $CD40^{high}$, and $IL\text{-}12^{+}$ monocyte-derived dendritic cells (moDC) from a range of healthy donors. **Step 1A,** Example heat maps show fold change values starting at 1.0 (no response) and ending at the median value observed for the positive control KLH. Results are indicated for monoclonal antibodies (mAb) with low, low/moderate, moderate, and high immunogenicity risk. **Step 1B,** Normalize median fold change values to 1.0 for each protein where 1.0 is the median value observed for the positive control KLH. The 'combined score' is the weighted average of the normalized values where each marker is weighted equally. **Step 2,** Use the combined score to rank proteins for immunogenic risk from 0 to 1: < 0.2 'low risk', 0.2 – 0.4 'low/moderate risk', 0.4 – 0.6 'moderate risk', and > 0.6 'high risk'. **Step 3,** Consider performing the Transwell migration test in a range of healthy donors to confirm the result. The Transwell migration test better captures moderate versus low/moderate risk. **Step 4,** For low to low/moderate scores, perform the Transwell migration test at the 'high' concentration gradient (i.e., 100 to 1000 μg/mL protein) and calculate the Migration Index by normalizing the percent migrated moDC toward protein + CXCL12 + CCL21 to the percent migrated moDC toward media + CXCL12 + CCL21. **Step 5,** Normalize the median Migration Index to 1.0 for each protein where 1.0 is the median value observed for the positive control KLH. The Transwell test confirms a result of low to low/moderate risk for trastuzumab and predicts a moderate risk for adalimumab. **Step 6,** For high and very high scores, the Transwell migration test can be performed at a 'low' concentration gradient (i.e., 10 to

50 μg/mL protein). Calculate the Migration Index as in **step 4**. **Step 7,** Normalize the median Migration Index as in **step 5**. Use the normalized Migration Index to confirm ranking of immunogenic risk: < 0.4 'low risk', 0.4 – 0.6 'moderate risk', and > 0.6 'high risk'. The Transwell test confirms a result of high risk for ATR-107 and HuA33. KLH, keyhole limpet hemocyanin.

*The median fold change over untreated is normalized to the median value of the positive control (KLH) data set.

❖The combined score is the weighted average of normalized scores for CXCR4, CD40, and IL-12, with equal weights for each marker.

†The Transwell migration test better captured moderate and low-moderate risk when compared to the 'combined score' from step 2.

Supplementary Tables and Table Legends

Supplementary Table 1. Characteristics of healthy donor sources for peripheral blood mononuclear cells

Donor	Age Range	HLA-A		HLA-B		HLA-C	
589	20 – 29	02:01	26:08	07:02	13:02	06:02	07:02
919	30 – 39	01:01	26:01	08:01	38:01	07:01/02	12:02/03
236	40 – 49	01:01	01:01	08:01	51:01	07:01	16:01/02
773	40 – 49	11:01	11:01	15:01	49:01	03:02/04/05	07:01/02/05
890	20 – 29	02:01	03:01	15:01	40:01	03:02/04/05/06	03:04/06/09
014	20 – 29	02:01	29:01/02	44:03	51:01	15:02/03/07	16:01/02/08
926	40 – 49	03:01	03:01	18:01	35:03	04:01	07:01
274	30 – 39	02:01	32:01	15:01	27:05	02:02	03:04
501	40 – 49	03:01	68:01	55:01	57:01	03:03	06:02

Donor information was provided by the vendor Cytologics LLC. *BMI* Body mass index, *HLA* Human leukocyte antigen.

Supplementary Table 2. Specifications for monoclonal antibodies

	Type of Molecule	Molecular Target	Route of Administration	ADA Incidence (%)	Reference
Tocilizumab	Humanized IgG1	IL-6R	Subcutaneous	0.8 - 2%	USPI (Actemra)
Rituximab	Chimeric murine/ human IgG1	CD20	Subcutaneous	2 - 12%	USPI (Rituxan Hycela)
Adalimumab	Human IgG1	TNFα	Subcutaneous	3 - 28%	USPI (Humira), Bartelds et al, 2011
Emicizumab	Humanized IgG4	FX and FIXa	Subcutaneous	5.10%	USPI (Hemlibra)
Trastuzumab	Humanized IgG1	HER2	Subcutaneous	16%	USPI (Herceptin Hylecta)
ATR-107	Human IgG1	IL-21R	Subcutaneous/ Intravenous	76%	Hua et al, 2013
HuA33	Humanized IgG1	A33	Intravenous	73%	Welt et al, 2003

The clinically reported anti-drug antibody incidence for monoclonal antibodies and their target, isotype, and route of administration. *ADA* Anti-drug antibody, *USPI* United States package insert
