# Supplementary material for: A mechanistic marker-based screening tool to predict clinical immunogenicity of biologics
Source: Commun Med (Lond). 2023 Dec 8;3:174. doi: 10.1038/s43856-023-00413-7 (PMC10709359; doi:10.1038/s43856-023-00413-7)
Supplement: Supplementary file 2 — Description of Additional Supplementary Files [file 43856_2023_413_MOESM2_ESM.pdf]

Description of Additional Supplementary Files

File Name: Supplementary Data
Description: Excel spreadsheet of underlying numerical data from all figures in the manuscript.
