# Supplementary material for: A mechanistic marker-based screening tool to predict clinical immunogenicity of biologics
Source: Commun Med (Lond). 2023 Dec 8;3:174. doi: 10.1038/s43856-023-00413-7 (PMC10709359; doi:10.1038/s43856-023-00413-7)
Supplement: Supplementary file 4 — Reporting Summary [file 43856_2023_413_MOESM4_ESM.pdf]

nature portfolio

Corresponding author(s): Sathy Balu-Iyer

Last updated by author(s): 11/4/2023

Reporting Summary

Nature Portfolio wishes to improve the reproducibility of the work that we publish. This form provides structure for consistency and transparency in reporting. For further information on Nature Portfolio policies, see our Editorial Policies and the Editorial Policy Checklist.

Statistics

For all statistical analyses, confirm that the following items are present in the figure legend, table legend, main text, or Methods section.

n/a	Confirmed	
☐	☒	The exact sample size (n) for each experimental group/condition, given as a discrete number and unit of measurement
☐	☒	A statement on whether measurements were taken from distinct samples or whether the same sample was measured repeatedly
☐	☒	The statistical test(s) used AND whether they are one- or two-sided<br>*Only common tests should be described solely by name; describe more complex techniques in the Methods section.*
☒	☐	A description of all covariates tested
☐	☒	A description of any assumptions or corrections, such as tests of normality and adjustment for multiple comparisons
☐	☒	A full description of the statistical parameters including central tendency (e.g. means) or other basic estimates (e.g. regression coefficient) AND variation (e.g. standard deviation) or associated estimates of uncertainty (e.g. confidence intervals)
☐	☒	For null hypothesis testing, the test statistic (e.g. F, t, r) with confidence intervals, effect sizes, degrees of freedom and P value noted<br>*Give P values as exact values whenever suitable.*
☒	☐	For Bayesian analysis, information on the choice of priors and Markov chain Monte Carlo settings
☒	☐	For hierarchical and complex designs, identification of the appropriate level for tests and full reporting of outcomes
☒	☐	Estimates of effect sizes (e.g. Cohen's d, Pearson's r), indicating how they were calculated

Our web collection on statistics for biologists contains articles on many of the points above.

Software and code

Policy information about availability of computer code

Data collection	Flow cytometry data was collected in BD FACS Diva
Data analysis	Commercially available software was used as mentioned in the manuscript

For manuscripts utilizing custom algorithms or software that are central to the research but not yet described in published literature, software must be made available to editors and reviewers. We strongly encourage code deposition in a community repository (e.g. GitHub). See the Nature Portfolio guidelines for submitting code & software for further information.

Data

Policy information about availability of data

All manuscripts must include a data availability statement. This statement should provide the following information, where applicable:

- Accession codes, unique identifiers, or web links for publicly available datasets
- A description of any restrictions on data availability
- For clinical datasets or third party data, please ensure that the statement adheres to our policy

All data are contained within the manuscript text, Supplementary Information, and Supplementary Data. Source data for all figures can be found in Supplementary Data.

Human research participants

Policy information about studies involving human research participants and Sex and Gender in Research.

Reporting on sex and gender	Not applicable
Population characteristics	Not applicable
Recruitment	Not applicable
Ethics oversight	Not applicable

Note that full information on the approval of the study protocol must also be provided in the manuscript.

Field-specific reporting

Please select the one below that is the best fit for your research. If you are not sure, read the appropriate sections before making your selection.

☒ Life sciences ☐ Behavioural & social sciences ☐ Ecological, evolutionary & environmental sciences

For a reference copy of the document with all sections, see nature.com/documents/nr-reporting-summary-flat.pdf

Life sciences study design

All studies must disclose on these points even when the disclosure is negative.

Sample size	All experiments were performed with triplicates to provide enough data points for statistical testing.
Data exclusions	No data was excluded from analysis.
Replication	Experiments were replicated in at least half of the donor cells tested.
Randomization	No experimental grouping was involved.
Blinding	Blinding was not possible for the cell culture experiments.

Reporting for specific materials, systems and methods

We require information from authors about some types of materials, experimental systems and methods used in many studies. Here, indicate whether each material, system or method listed is relevant to your study. If you are not sure if a list item applies to your research, read the appropriate section before selecting a response.

Materials & experimental systems

n/a	Involved in the study	
☐	☒	Antibodies
☒	☐	Eukaryotic cell lines
☒	☐	Palaeontology and archaeology
☒	☐	Animals and other organisms
☒	☐	Clinical data
☒	☐	Dual use research of concern

Methods

n/a	Involved in the study	
☒	☐	ChIP-seq
☐	☒	Flow cytometry
☒	☐	MRI-based neuroimaging

Antibodies

Antibodies used	Research grade biosimilar antibodies from Bio X Cell (adalimumab SIM0001, trastuzumab SIM0005, and rituximab SIM0008, and from Absolute Antibody (anti-A33 HuA33 AB00782-10.0, anti-IL-21R ATR-107 AB01293-10.0, and anti-IL-6R tocilizumab AB00737-10.0)
Validation	Manufacturers' websites state that the antibodies were produced using structural sequences of the marketed monoclonal antibodies and provided target binding specificity.

Flow Cytometry

Plots

Confirm that:

☒ The axis labels state the marker and fluorochrome used (e.g. CD4-FITC).

☒ The axis scales are clearly visible. Include numbers along axes only for bottom left plot of group (a 'group' is an analysis of identical markers).

☒ All plots are contour plots with outliers or pseudocolor plots.

☒ A numerical value for number of cells or percentage (with statistics) is provided.

Methodology

Sample preparation	Monocyte-derived dendritic cells were collected from cell culture and washed with PBS before staining.
Instrument	BD LSRFortessa
Software	Flow cytometry data was collected in BD FACS DIVA and analyzed in Flow Jo v10.7
Cell population abundance	Monocyte-derived dendritic cells represented approximately 70-90% of the total cell population as determined by staining for HLA-DR, CD11c, and DC-SIGN.
Gating strategy	Total cell events were gated based on FSC-A vs SSC-A then single cell events were gated on FSC-A vs FSC-H. Live cells were gated based on low expression of viability ghost dye (below about 100 MFI), and monocyte-derived DCs were gated based on positive expression of CD11c and HLA-DR, simultaneously.

☒ Tick this box to confirm that a figure exemplifying the gating strategy is provided in the Supplementary Information.
